# Refusal Rates to Organ Donation in Intensive Care Units Among Immigrant Populations in Italy

**DOI:** 10.3389/ti.2023.11674

**Published:** 2023-09-07

**Authors:** Alessandra Agnese Grossi, Francesca Puoti, Umberto Maggiore, Massimo Cardillo

**Affiliations:** ^1^ Center for Clinical Ethics, Department of Biotechnologies and Life Sciences, University of Insubria, Varese, Italy; ^2^ Department of Human Sciences, Innovation and Territory, University of Insubria, Como, Italy; ^3^ Italian National Transplant Center (CNT), Istituto Superiore di Sanità, Rome, Italy; ^4^ Nephrology Unit, Dipartimento di Medicina e Chirurgia, Università di Parma, Parma, Italy

**Keywords:** organ donation, organ transplantation, migrants, immigrants, ethnic minorities

Dear Editors,

Immigration is an ongoing and emergent phenomenon worldwide. With 82 million subjects who have migrated from other countries, Europe is currently first at the global level as host of international migrants [1]. In Europe, Italy stands third in the number of regular residents from outside the European Union (EU) (5.2 million). Over the past 20 years, Italy has become an increasingly diverse country. Given the recency of the immigration phenomenon, adult immigrant and ethnic minority groups in Italy coincide. Ethnic minorities originate mostly from Eastern Europe (Romania, Albania, Ukraine, and Moldova), Northern Africa (Morocco and Egypt), Asia (China) and South Asia (India, Philippines, Bangladesh and Pakistan) [2], with Eastern European populations having the longer-standing immigration history.

It is well known that the attitude toward post-mortem organ donation is complex and multifactorial and may be influenced by many intersecting factors requiring a socio-ecological approach (i.e., considering the factors intervening at the individual, interpersonal and societal levels) to enable understanding. This is especially relevant when it comes to considering the factors underlying the attitude of individuals who have migrated from other countries or who are from ethnic minorities. At the individual level, many factors may play a role. These factors include cultural and religious beliefs, language proficiency, socioeconomic status, and low health literacy. Further, lack of knowledge of organ donation and transplantation, lack of trust toward the healthcare system and healthcare professionals (HCP), and lack of familiarity with the complexity of the healthcare system may be influential. Additional factors may include also the individual reasons for and circumstances of migration, time elapsed since immigration as a potential proxy for acculturation and/or integration, and organ trafficking or corruption in healthcare systems being widespread practices in countries of origin. Besides, HCPs’ lack of training for the development of intercultural competences and communicative/relational abilities, and interpersonal dynamics between potential donors’ families and HCPs in intensive care units (ICU) may equally account for a portion of attitudes among immigrant donors’ families in ICUs. At the healthcare system level, the inability of institutional actors to communicate about organ donation and transplantation in a culturally sensitive fashion among the public and to reach out effectively to these communities by improved participation and involvement of local faith/community representatives may equally play a role. Availability of culturally competent and, more broadly, diversity sensitive healthcare services, administrative difficulties, poor care coordination among the actors involved, and other features at organizational, local levels and, finally, the broader societal context (i.e., integration and migration policies, discrimination) also have the potential to stand among the contributing factors [3].

We have extracted data on 24,222 donors between 1 January 2012 and 31 December 2021 from the Transplant Information System (SIT) of the Italian National Transplant Center (CNT). As in earlier studies [4, 5], non-EU-born individuals were categorized as Eastern European-born and non-European-born as distinguished from EU-born (see the Supplementary Appendix for additional details) and included groups that were represented by at least 30 subjects. Out of 24,222 donors, 1,077 (4.4%) were non-EU-born and 1,771 (7.3%) were foreign-born. We estimated refusal rates along with 95% credible intervals (95% CI) based on Bayesian logistic models that was adjusted for Italian region of donation (The Stata and R Stan code for the statistical analyses are freely available at^1^; see the Supplementary Appendix for more details on the size of each group and on the distribution across Italian region of donation). As shown in Figure 1, refusal rates varied greatly according to both donor’s ethnicity (Figure 1A), and donor’s country of birth (Figure 1B). Compared to the native-born Italian population, the refusal rates were higher across virtually every immigrant group and mostly originating from non-Western countries, with the exception of individuals from Sri Lanka. The mean probability of refusal ranged from 27.2% (95% CI: 18.5%–42.3%) for EU-born donors to 69% (95% CI: 55.5–83.0) for North African and Middle-Eastern donors (Figure 1A); from 24.3% (95% CI: 14.5%–37.0%) for donors from Poland to 75.5 (95% CI: 64.9–84.0) for donors from China (Figure 1B). Studies on refusal rates among individuals who have migrated from other countries are scarce in Europe. To the best of our knowledge, there are only two reports, one from the UK and the other from Norway that have examined refusal rates according to ethnicity and/or immigration status. Consistent with our findings, they have both reported higher refusal rates among migrant and ethnic minority individuals relative to their native-born or White referents [6, 7]. However, the UK report was unable to present the detailed data of refusal rates according to countries of origin and referred only to broad ethnicity categories (i.e., Black, Asian and other minority ethnic groups) [6]. In contrast, the Norwegian article did not provide any data of either ethnicity or immigrant status as these data are not routinely collected in Norway. Therefore, the article only provided a general overview of the problem [7].

**FIGURE 1:**
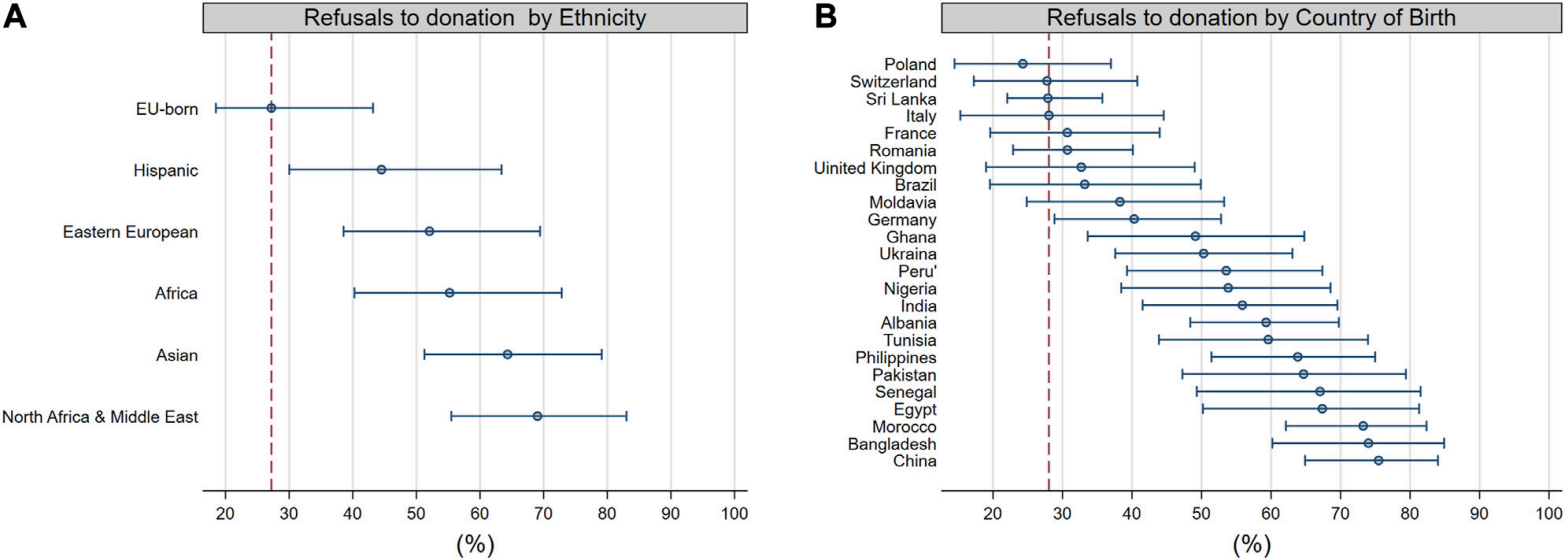
Proportion of refusals to donation according to ethnicity **(A)** and country of birth **(B)**. The circles represent the mean, the horizontal bars the 95 percent credible intervals. A dotted vertical bar is drawn at the mean of the referent group, namely, EU-Born **(A)** and Italy **(B)**. A 95% percent credible interval that does not cross the vertical red bars suggests a low probability (i.e., less than 5%) that the refusal propensity of the group is alike the referent group. We included only groups that were represented by at least 30 subjects. The plots results from fitted Bayesian regression models that were adjusted for Italian region of donation.

We contend that it is an ethical duty and responsibility to foster shared decision-making (i.e., “a relational process … *allowing* decisions to develop over time by jointly respecting clinical indications and individual … subjective considerations, values, needs, preferences, life circumstances and goals”) [8] to enable free and informed choices surrounding organ donation among these populations. Consideration of the informative, cultural, and psychosocial needs of these communities in relation to deceased organ donation and end-of-life care; understanding of the difficulties and educational gaps among HCPs and of the deficiencies at the organizational level allowing to communicate in a culturally sensitive fashion with bereaved family members in ICUs, and the promotion of an ongoing dialogue among all stakeholders (including representatives from minority, migrant and faith communities) are critical for the subsequent development of interventions directed towards these populations [9, 10]. The CNT has recently initiated a project named Fostering And Improving equity, participation and inclusion in Transplantation Healthcare (FAITH) to address the existing gaps in the entire organ donation and transplantation pathway (i.e., promotion of the culture of organ donation and transplantation among the public/communities, relational processes in ICUs, and relational/educational processes at transplant centers with transplant recipients and, when this applies, their potential living donors) to enable the implementation of shared decision-making across the entire process [9]. Future studies will examine whether simultaneous interventions on the modifiable factors at the different levels may improve the ability of the transplantation system to respond effectively to the needs of an increasingly diverse and multicultural society.

## Data Availability

The datasets presented in this study can be found in online repositories. The names of the repository/repositories and accession number(s) can be found in the article/Supplementary Material.

## Footnotes

1
https://github.com/UMaggiore/Refusal-Rates

## Abbreviations

CNT, Italian National Transplant Center; EU, European Union; HCP, Healthcare Professional; ICU, Intensive Care Unit.
