# Supplementary material for: Refusal Rates to Organ Donation in Intensive Care Units Among Immigrant Populations in Italy
Source: Transpl Int. 2023 Sep 7;36:11674. doi: 10.3389/ti.2023.11674 (PMC10513101; doi:10.3389/ti.2023.11674)
Supplement: Supplementary file 1 [file DataSheet1.pdf]

Supplementary Appendix

Data collection and definitions
We have extracted data on 24,222 donors between 1 January 2012-31 December 2021 from the Transplant Information System (SIT) of the Italian National Transplant Center (CNT).

As in earlier studies (1,2), non-EU-born individuals were categorized as Eastern European-born and non-European-born as distinguished from EU-born. Eastern European-born patients included individuals from Albania, Moldavia, former Yugoslavian countries, Ukraine and other countries of the Eastern European and Balkans area. Non-European-born patients included individuals from Asian (South-East and North-East Asia), Latin American (Hispanic), Northern African and Middle Eastern (Northern-Africa and Middle-East) and Sub-Saharan African countries (African) (1,2).

A ‘migrant’ is “any person who is outside a State of which he or she is a citizen or national, or, in the case of a stateless person, his or her State of birth or habitual residence. The term includes migrants who intend to move permanently or temporarily, and those who move in a regular or documented manner as well as migrants in irregular situations” (3). The immigrant category excludes persons who travel for tourism or business purposes and excludes intra-EU mobility (4).

Similarly, but still, differently, ‘ethnicity’ is “the social group a person belongs to, and either identifies with or is identified with by others, as a result of a mix of cultural and other factors including language, diet, religion, ancestry, and physical features” that are shared by individuals in the same group (5).

Although we were unable to identify and exclude tourists and persons travelling to Italy for business purposes, it is likely that only few if any were included in our database.

Statistical analyses
We estimated refusal rates along with 95% credible intervals (95%CI) based on Bayesian logistic models with Italian region of donation and ethnicity or country of origin fitted as crossed random effects. We included only groups with at least 30 subjects. We used Bayesian analysis because, compared to standard fixed-effects frequentist analysis, it more easily deals with crossed-effects (e.g., same country of origin nested within different regions of donation), and has a better small sample inference. The Stata and R Stan code for the statistical analyses are freely available at https://github.com/UMaggiore/Refusal-Rates.

Ethics statement
The retrospective analysis of the original data presented in this work was approved by the CNT and included data that were already anonymized and de-identified in the SIT database before extraction for the analysis. Therefore, the subjects could not be identified and, according to Italian legal regulations (D.L. 196/2003, art. 110-24 July 2008, art. 13), the study did not require Ethics Committee approval. The study was carried out in compliance with the ethical principles of the Declaration of Helsinki (with amendments).

Distribution of donors according to procurement region, ethnicity, and country of origin.
Out of 24,222 donors, 1,077 (4.4%) were non-EU and 1,771 (7.3%) were foreign-born. The frequency of donors according to region, ethnicity, and country of origin are reported in **Table S1**, **Table S2**, and **Table S3**. The distributions of ethnicity and country of origin according to the regions are reported in **Figure S1** (**Panel A**, **B, C**)

References

1. Grossi AA, Puoti F, Fiaschetti P, Di Ciaccio P, Maggiore U, Cardillo M. Kidney transplantation and withdrawal rates among wait-listed first-generation immigrants in Italy. Eur J Public Health. 2022 Jun 1;32(3):372–8.
2. Grossi AA, Maggiore U, Puoti F, Grossi PA, Picozzi M, Cardillo M. Association of immigration background with kidney graft function in a publicly funded health system: a nationwide retrospective cohort study in Italy. Transpl Int. 2020;33(11):1405–16.
3. International Organization for Migration (IOM). Glossary on Migration [Internet]. 2019. Available from: https://publications.iom.int/system/files/pdf/iml_34_glossary.pdf
4. European Commission. “Migrant” definition [Internet]. Available from: https://home-affairs.ec.europa.eu/pages/glossary/migrant_en
5. Bhopal R. Glossary of terms relating to ethnicity and race: for reflection and debate. J Epidemiol Community Health. 2004;58(6):441–5.

Table S1. Frequency of donors according to region

Donors' Region of residency	Number
ABRUZZO	401
BASILICATA	182
CALABRIA	582
CAMPANIA	1311
EMILIA ROMAGNA	2174
FRIULI VENEZIA GIULIA	594
LAZIO	2230
LIGURIA	513
LOMBARDIA	3911
MARCHE	728
MOLISE	57
PIEMONTE	2273
PROVINCIA AUTONOMA DI BOLZANO	108
PROVINCIA AUTONOMA DI TRENTO	174
PUGLIA	984
SARDEGNA	680
SICILIA	1413
TOSCANA	3293
UMBRIA	219
VALLE D'AOSTA	52
VENETO	2343
Total	24222

Table S2. Frequency of donors according to ethnicity

Donors' ethnicity	Number
EU-BORN	23145
EASTERN EUROPEAN	301
ASIAN	379
HISPANIC	95
AFRICAN	121
NORTH AFRICA & MIDDLE EAST	181
Total	24222

Table S3. Frequency of donors according to country of origin

Donors' country of origin	Number
ALBANIA	160
BANGLADESH	50
BRAZIL	37
CHINA	115
EGYPT	32
PHILIPPINES	92
FRANCE	70
GERMANY	103
GHANA	44
INDIA	57
ITALY	22451
MOROCCO	105
MOLDAVIA	53
NIGERIA	47
PAKISTAN	31
PERU'	58
POLAND	65
UNITED KINGODM	38
ROMANIA	346
SENEGAL	30
SRI LANKA	34
SWITZERLAND	72
TUNISIA	44
UKRAINE	88
**Total**	24222

Supplementary Figure S1

A

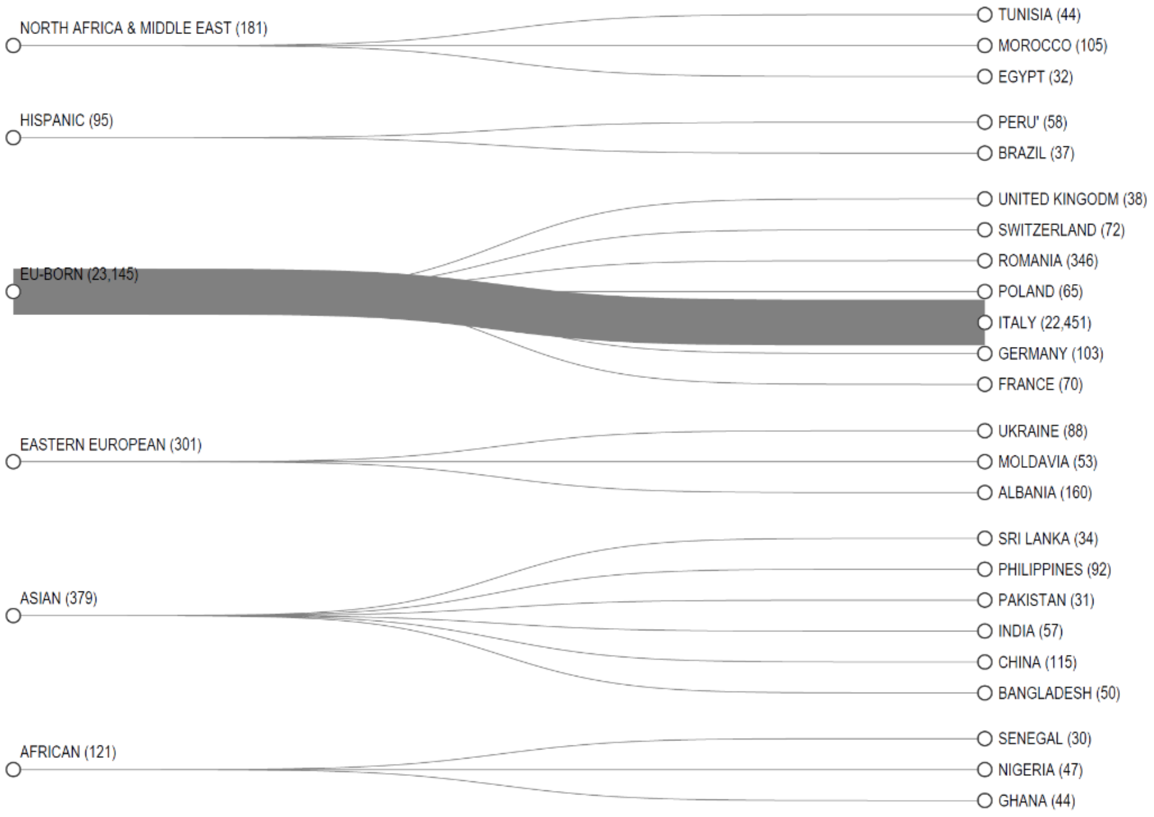

B

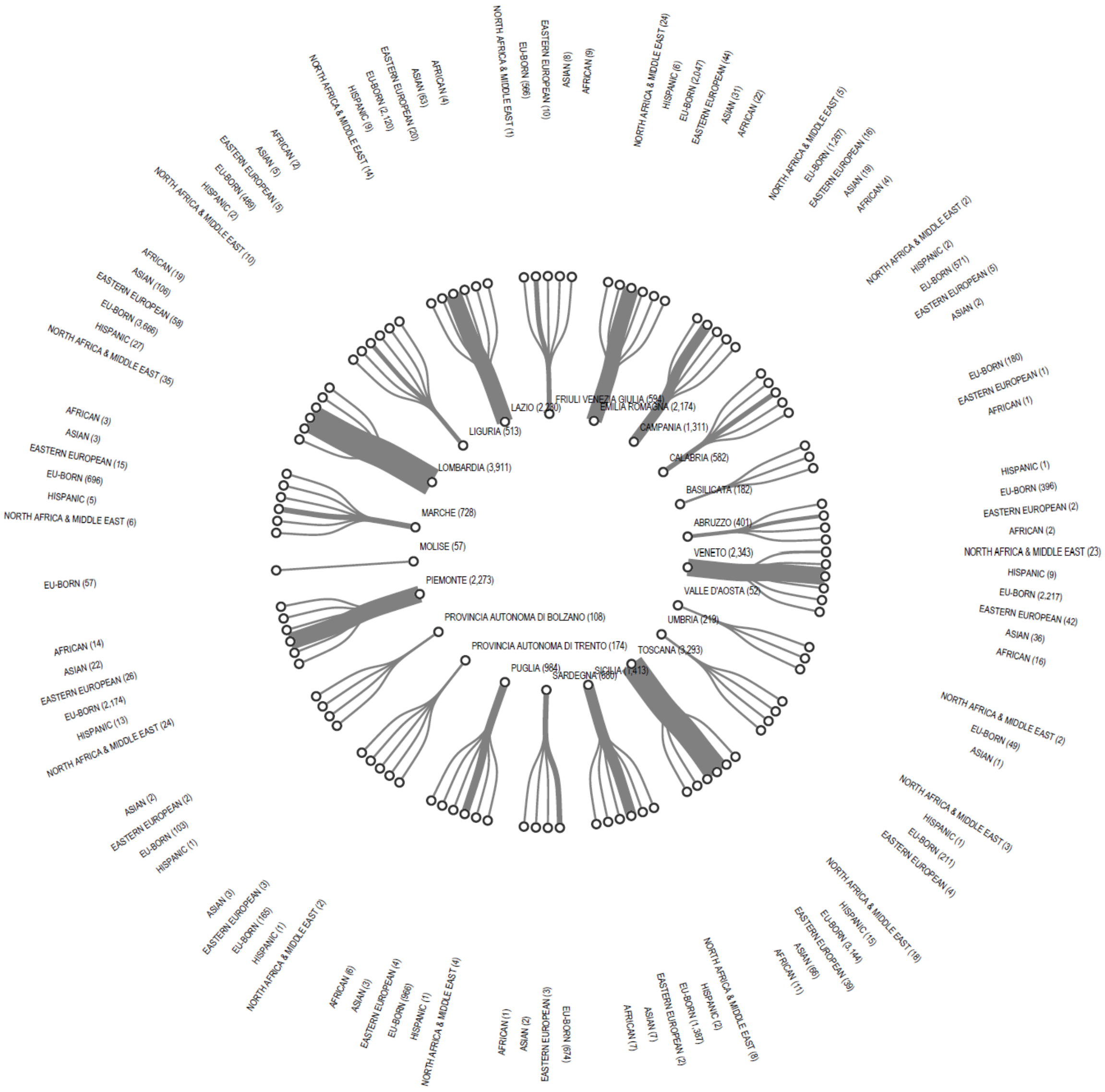
LAZIO (2,230)
FRIULI VENEZIA GIULIA (594)
EMILIA ROMAGNA (2,174)
CAMPANIA (1,311)
CALABRIA (582)
BASILICATA (182)
ABRUZZO (401)
VENETO (2,343)
VALLE D'AOSTA (52)
UMBRIA (219)
TOSCANA (3,293)
SICILIA (7,413)
SARDEGNA (686)
PUGLIA (984)
PROVINCIA AUTONOMA DI TRENTO (174)
PROVINCIA AUTONOMA DI BOLZANO (108)
PIEMONTE (2,273)
MOLISE (57)
MARCHE (728)
LOMBARDIA (3,911)
LIGURIA (513)
NORTH AFRICA & MIDDLE EAST (1)
EU-BORN (566)
EASTERN EUROPEAN (10)
ASIAN (8)
AFRICAN (9)
NORTH AFRICA & MIDDLE EAST (24)
HISPANIC (6)
EU-BORN (2,047)
EASTERN EUROPEAN (44)
ASIAN (31)
AFRICAN (22)
NORTH AFRICA & MIDDLE EAST (5)
EU-BORN (1,267)
EASTERN EUROPEAN (16)
ASIAN (19)
AFRICAN (4)
NORTH AFRICA & MIDDLE EAST (2)
HISPANIC (2)
EU-BORN (571)
EASTERN EUROPEAN (5)
ASIAN (2)
EU-BORN (180)
EASTERN EUROPEAN (1)
AFRICAN (1)
HISPANIC (1)
EU-BORN (396)
EASTERN EUROPEAN (2)
AFRICAN (2)
NORTH AFRICA & MIDDLE EAST (23)
HISPANIC (9)
EU-BORN (2,217)
EASTERN EUROPEAN (42)
ASIAN (36)
AFRICAN (16)
NORTH AFRICA & MIDDLE EAST (2)
EU-BORN (49)
ASIAN (1)
NORTH AFRICA & MIDDLE EAST (3)
HISPANIC (1)
EU-BORN (211)
EASTERN EUROPEAN (4)
NORTH AFRICA & MIDDLE EAST (18)
HISPANIC (15)
EU-BORN (3,144)
EASTERN EUROPEAN (39)
ASIAN (86)
AFRICAN (11)
NORTH AFRICA & MIDDLE EAST (8)
HISPANIC (2)
EU-BORN (1,387)
EASTERN EUROPEAN (2)
ASIAN (7)
AFRICAN (7)
EU-BORN (674)
EASTERN EUROPEAN (3)
ASIAN (2)
AFRICAN (1)
NORTH AFRICA & MIDDLE EAST (4)
HISPANIC (1)
EU-BORN (966)
EASTERN EUROPEAN (4)
ASIAN (3)
AFRICAN (6)
NORTH AFRICA & MIDDLE EAST (2)
HISPANIC (1)
EU-BORN (165)
EASTERN EUROPEAN (3)
ASIAN (3)
HISPANIC (1)
EU-BORN (103)
EASTERN EUROPEAN (2)
ASIAN (2)
NORTH AFRICA & MIDDLE EAST (24)
HISPANIC (13)
EU-BORN (2,174)
EASTERN EUROPEAN (26)
ASIAN (22)
AFRICAN (14)
EU-BORN (57)
NORTH AFRICA & MIDDLE EAST (6)
HISPANIC (5)
EU-BORN (696)
EASTERN EUROPEAN (15)
ASIAN (3)
AFRICAN (3)
NORTH AFRICA & MIDDLE EAST (35)
HISPANIC (27)
EU-BORN (3,666)
EASTERN EUROPEAN (58)
ASIAN (106)
AFRICAN (19)
NORTH AFRICA & MIDDLE EAST (10)
HISPANIC (2)
EU-BORN (486)
EASTERN EUROPEAN (5)
ASIAN (5)
AFRICAN (2)
NORTH AFRICA & MIDDLE EAST (14)
HISPANIC (9)
EU-BORN (2,120)
EASTERN EUROPEAN (20)
ASIAN (63)
AFRICAN (4)

C

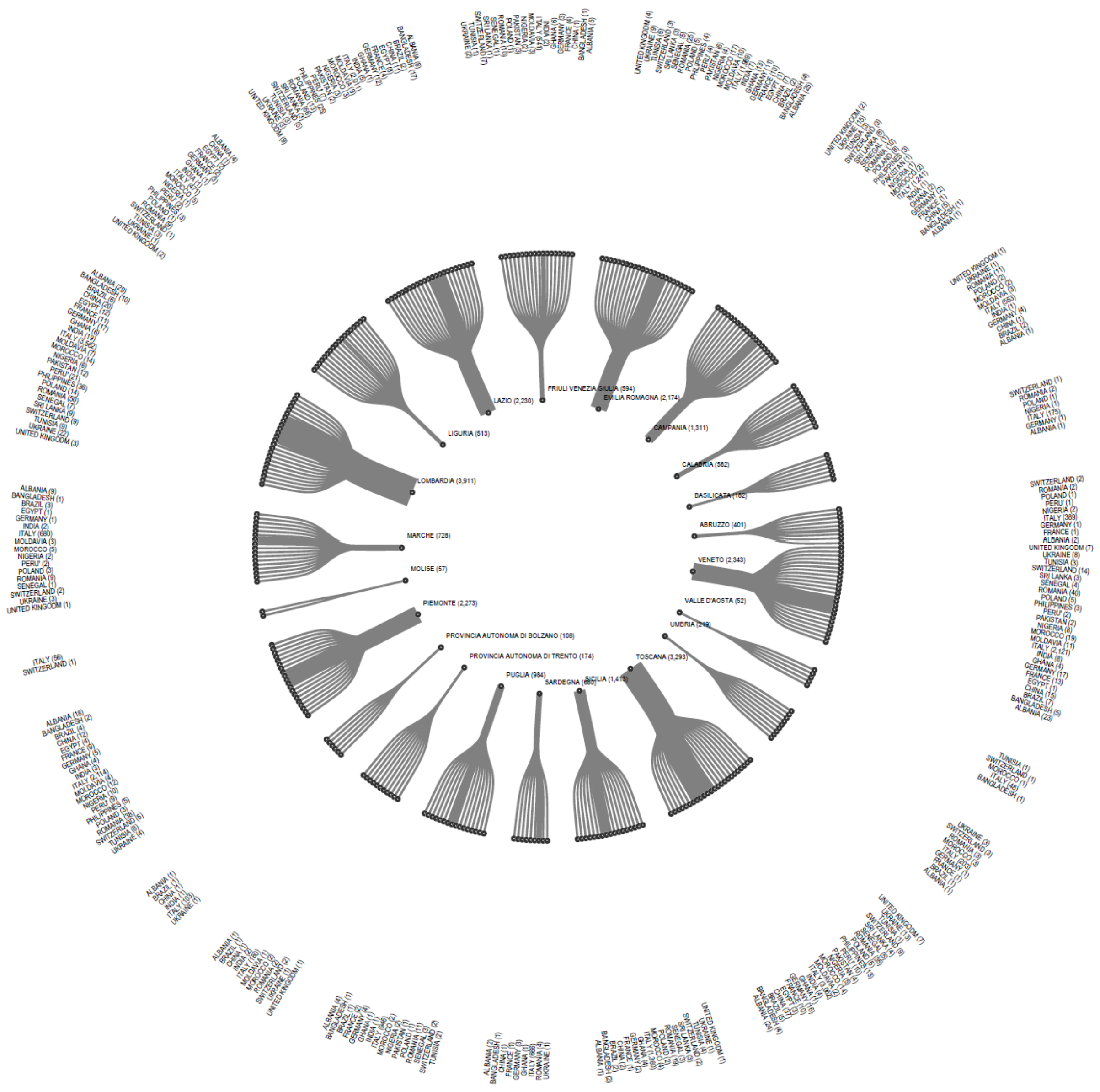

Supplementary Figure S1. Clustered tree plots showing the classification and frequency according to Ethnicity and Country of Birth (Panel A), the frequency distribution of Ethnicity across Italian regions (Panel B), and the distribution of Country of Birth across Italian regions (Panel C).
